# Effects of Self-Esteem on Self-Viewing: An Eye-Tracking Investigation on Mirror Gazing

**DOI:** 10.3390/bs11120164

**Published:** 2021-11-29

**Authors:** Jonas Potthoff, Anne Schienle

**Affiliations:** Institute of Psychology, University of Graz, 8010 Graz, Austria; anne.schienle@uni-graz.at

**Keywords:** face, self-perception, mirror, eye-tracking, self-esteem, self-disgust, narcissism

## Abstract

While some people enjoy looking at their faces in the mirror, others experience emotional distress. Despite these individual differences concerning self-viewing in the mirror, systematic investigations on this topic have not been conducted so far. The present eye-tracking study examined whether personality traits (self-esteem, narcissism propensity, self-disgust) are associated with gaze behavior (gaze duration, fixation count) during free mirror viewing of one’s face. Sixty-eight adults (mean age = 23.5 years; 39 females, 29 males) viewed their faces in the mirror and watched a video of an unknown person matched for gender and age (control condition) for 90 s each. The computed regression analysis showed that higher self-esteem was associated with a shorter gaze duration for both self-face and other-face. This effect may reflect a less critical evaluation of the faces.

## 1. Introduction

Looking at one’s face in the mirror can evoke strong feelings. A prominent example from Greek mythology is Narcissus, who fell in love with his own image reflected in a pool of water. However, in everyday life, many people find it distressing to look at themselves [1,2]. For example, in a study by Barnier and Collison [3], five minutes of mirror gazing decreased satisfaction with one’s appearance, perceived attractiveness, and self-esteem. Such adverse effects were especially prominent in those observers who had been dissatisfied with their appearance already before the mirror viewing [4].

An interesting question is: how do people view themselves in the mirror? Elaborate measures such as eye-tracking can help to answer this question. To the best of our knowledge, no eye-tracking studies have been conducted before on the viewing of one’s face in a mirror, which, therefore, was the focus of the present study. Previous investigations have examined gaze behavior toward photos or videos depicting self-face vs. other-face [5,6,7,8,9,10]. These studies showed that the “own face” (compared to the faces of others) captures attention. The own face is identified faster among other faces and better recalled [9,11]. Additionally, self-faces are explored differently than unfamiliar faces during free viewing (e.g., more fixations are directed on the eyes of other faces than the self-face [8]). Finally, self-faces can distract attention from a task. In a study by Devue et al. [9], participants searched for a target face (e.g., with an open mouth) among distractor faces (e.g., with a closed mouth). Self-face distractors were fixated longer than other-face distractors.

The inspection of the own face (in pictures) is influenced by different personality traits, such as self-disgust and self-esteem. Self-disgust involves a lasting appraisal of aspects of the self as disgusting [12]. Ypsilanti, Gettings et al. [5] presented participants with picture pairs consisting of their face and an unknown face. Those participants with high self-disgust showed attentional avoidance of their faces (reduced gaze duration). In the second study of this research group, Ypsilanti, Robson et al. [6] focused on maintaining visual attention vs. gaze avoidance of the own face. Again, high self-disgust was associated with avoidance of the own face. The participants shifted their gaze over the trial duration away from their face toward the unknown face. These findings suggest that dissatisfaction with one’s visual appearance is associated with a reduced viewing time of one’s face.

Additionally, the personality trait self-esteem (confidence and satisfaction in oneself) is related to self-viewing behavior. In a study by Hu et al. [13], participants high vs. low in self-esteem showed a greater pupil size while looking at their face compared to an unfamiliar face. Participants with high self-esteem also identified their faces faster than unknown faces [13].

Inflated self-esteem, narcissism, might also play a role in the way people view their faces. Narcissism is characterized by an overestimation of one’s capabilities, combined with a high need for admiration by others, perception of uniqueness, lack of empathy, and a tendency to take advantage of others [14]. People with high narcissism propensity (NP) enjoy watching themselves more than people with low NP. In a study by Robins and John [15], participants were asked whether they would prefer watching themselves or others in a videotaped group exercise. Participants with high scores on the Narcissistic Personality Inventory (NPI; [16]) chose to view themselves more often instead of choosing to view someone else. Furthermore, participants high in NP reported more frequent mirror viewing than participants with low NP. In a brain imaging study, self-face viewing was associated with increased activation in the anterior cingulate cortex (ACC) in participants with high NP [17]. The authors interpreted the ACC activation as an indicator of negative affect. The elicitation of negative emotions reflects that looking at the own face can challenge ego inflation [18]. The results of this investigation suggest that vulnerable aspects of narcissism may not be observable in self-report studies. Therefore, it is necessary to reevaluate the assumption that narcissists look at themselves in the mirror more extensively than individuals with low-level narcissism [15].

It has to be noted that viewing oneself in a picture vs. a mirror differs in many ways. First, mirror viewing is dynamic and can be adjusted based on visual feedback. Second, dynamic faces are perceived as more attractive than static faces [19]. Third, when we look at a mirror, the left side of our face is reflected on the right side. Since we are used to this mirrored self-depiction, we tend to perceive our faces more favorably in a mirror than in a photo [20,21].

The present study aimed at investigating associations between personality traits (self-disgust, self-esteem, narcissism propensity), and gaze parameters (gaze duration, fixation counts) during self-inspection of one’s face in the mirror. As the control stimulus, we used a video of the face of an unknown person (matched for age and gender) to compare the gaze parameters between self-face and other-face free viewing (for 90 s each).

We predicted that participants high in self-disgust would show conscious gaze avoidance of their faces (a shorter gaze duration) and a lower number of fixations as an indicator of less detail-exploring gaze behavior (i.e., fewer fixations = fewer details or locations are viewed) [5,6,22]. In contrast, high self-esteem and narcissism propensity should be associated with the opposite pattern, prolonged gaze [13,15], and an increased number of fixations (a more detailed viewing style).

An exploratory research question addressed possible gender differences in self-face viewing. To the best of our knowledge, there is no published eye-tracking study on this topic. However, there is some evidence that females tend to be more critical of their physical appearance than males [23,24]. This gender difference might contribute to differences in self-face processing. Because of the lack of eye-tracking investigations in this field, the present study conducted an exploratory comparison between female and male participants’ self-face and other-face viewing behavior.

## 2. Materials and Methods

### 2.1. Sample

This study was conducted with 75 participants (aged between 18 and 35 years), who reported normal or corrected-to-normal vision. We excluded seven participants because the eye-tracker recorded eye movements less than 50% of the time or calibration was impossible due to reflections on participants’ glasses. The final sample (*n* = 68) had a mean age of 23.5 years (*SD* = 3.8). Males (*n* = 29) and females (*n* = 39) did not differ in mean age (*p* = 0.14). Sixty-one participants (90%) were university students. The other participants were white-collar workers. The study was approved by the ethics committee of the University (ethical approval code: 39/104/63 ex 2019/20) and conducted according to the Declaration of Helsinki. All participants provided written informed consent.

### 2.2. Procedure

The participants were invited to an online survey that assessed demographic data (age, gender, occupation, educational status), exclusion criteria (reported diagnoses of somatic illness, mental disorders, psychotropic medication), and personality traits (self-disgust, self-esteem, narcissism propensity).

**Figure 1:**
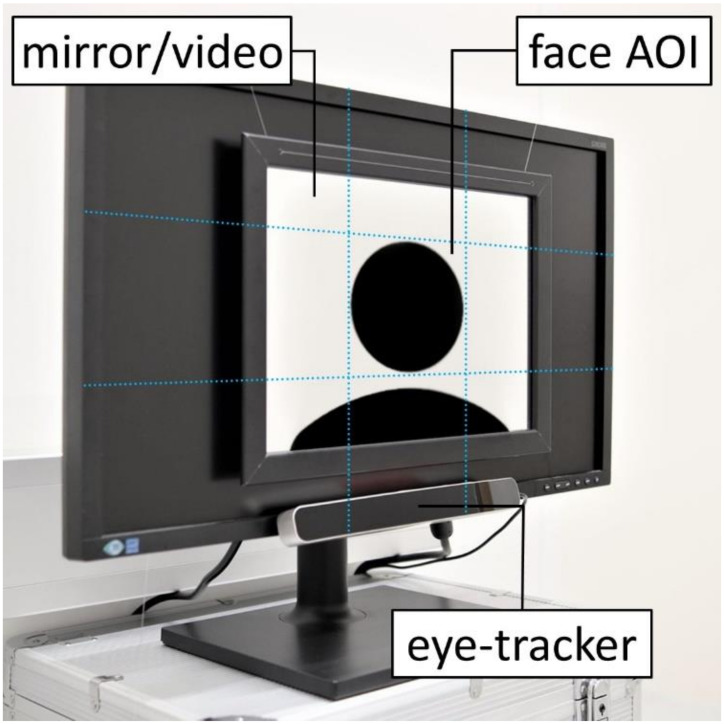
Hardware setup with mirror attached to the computer screen. Blue lines indicate Area of Interest (AOI) determination for self-face and other-face.

In the eye-tracking experiment, the participants freely viewed themselves in a mirror (attached to the screen of the computer; Figure 1) and watched a video of an unknown person displayed at the computer screen (same gender, similar age, neutral facial expression, no head movements, no speaking) for 90 s each (mirror/video: 39 × 29 cm; viewing distance: 60 cm) in a lit room (illuminance ~250 lux). The lighting enabled the participants to see their faces clearly in the mirror. The vertical and horizontal extent of the self-face and other-face were marked as perceived by two experimenters (see Figure 1). While one experimenter looked at his face in the mirror, another experimenter moved a ruler upward from the edge of the mirror until the ruler reached the reflection of the chin (above the chin rest). This position was defined as the lower end of the face and marked on the edge of the monitor with removable markers. The same procedure was repeated for the upper, right, and left borders of the face. The size and location of the face in the video were matched (resized and cropped) to the mirror condition. Areas of interest (AOIs) were determined in SMI BeGaze based on these outlines for self-face and other-face (same size of self-face and other-face AOI; Figure 1). The chinrest was positioned at the same height for all participants to position the self-face within the defined AOI. The trial order was randomized. Before each trial (when attaching/removing the mirror), the participants closed their eyes. At the beginning of each trial, the participants were asked to open their eyes. Keeping the eyes closed (instead of looking at a fixation cross before the trial started) ensured that both stimuli were visible for the same duration. The whole eye-tracking experiment took ap-proximately 10 min (informed consent <5 min; eye-tracker calibration ~2 min; 2 trials: 90 s per trial, intertrial interval ~30 s).

The paradigm was preregistered; data are available online: https://osf.io/fmv5s/ (accessed on 10 August 2021).

### 2.3. Eye Movement Recording and Analysis

We recorded eye movements with an SMI RED250mobile eye-tracker. Eye movements were defined by the standard SMI thresholds (saccade velocity-threshold: 40°/s; minimum fixation duration 50 ms). We analyzed gaze duration (i.e., total fixation duration) and fixation count within both face AOIs (self-face, other-face).

### 2.4. Questionnaires

The Narcissistic Personality Inventory (NPI) has been developed by Raskin and Hall [25] and is one of the most widely used personality measures for non-clinical levels of the trait narcissism. A German (NPI-D-17) version with 17 items has been validated by von Collani [26]. Typical items are “I think I am a special person”, “I will never be satisfied until I get all that I deserve.” or “I will usually show off if I get the chance.” which are rated on 5-point scales (1 = “does not apply”, 5 = “completely applies”). Cronbach’s alpha in the present sample was 0.90.

The Questionnaire for the Assessment of Self-Disgust (QASD; [12]) measures personal (devaluation of one’s physical appearance; 9 items; e.g., “I find myself repulsive”; 5-point scales: 0 = “not true at all”, 4 = “absolutely true”) and behavioral self-disgust (devaluation of one’s behavior; 5 items; e.g., “Some of my behaviors are repulsive to others”). The two self-disgust scores displayed high intercorrelation (*r* = 0.78) in the present sample, thus we used a global measure of self-disgust (encompassing all QASD items; α = 0.91). The same approach was used in previous research [27].

The Rosenberg Self-Esteem Scale was developed by Rosenberg in 1965 [28] and is a measure of global self-esteem (e.g., “On the whole, I am satisfied with myself”). In the present study, self-esteem was assessed via the German version of the Rosenberg Self-Esteem Scale, which was validated by Ferring and Filipp (RSES; [29]; α = 0.84; 10 items: e.g., “I am able to do things as well as most other people”; 4-point scales: 0 = “does not apply at all”; 3 = “completely applies”).

### 2.5. Statistical Analysis

First, Pearson correlations were calculated to investigate associations between the personality traits (self-esteem, narcissism propensity, self-disgust,) and gaze behavior (gaze duration, fixation count). Then, multiple regression analyses (simultaneous en-try/enter method) estimated the relationship between the traits and gaze behavior (view-ing of self/others). A mixed ANOVA tested the effects of Gender (male, female) and Condition (self-face, other-face) on total gaze duration and fixation count. If Mauchly’s test suggested a violation of sphericity, the Greenhouse–Geisser correction was applied. Post-hoc pairwise comparisons were Bonferroni–Holm corrected. We computed t-tests to compare questionnaire scores between males and females.

## 3. Results

### 3.1. Correlation Analyses

Pearson correlations between personality traits and gaze duration (self-face and other-face) are depicted in Table 1. Reported self-esteem correlated negatively with gaze duration (self-face and other-face) and positively with fixation count (other-face).

### 3.2. Regression Analyses

The regression for gaze duration on self-face was significant (*F*(3,64) = 4.45; *p* = 0.007; *R*^2^ = 0.173). Self-esteem was a significant predictor of gaze duration (Table 2). Higher self-esteem was associated with a shorter gaze duration. Also, for the gaze duration on the other-face the regression was significant (*F*(3,63) = 3.42; *p* = 0.022; *R*^2^ = 0.140) with higher self-esteem predicting a shorter gaze duration.

For the fixation count, the regression model was not significant for self-face (*F*(3,64) = 1.41; *p* = 0.247; *R*^2^ = 0.062) and other-face (*F* (3,63) = 1.91; *p* = 0.137, *R*^2^ = 0.083).

### 3.3. Exploratory Analyses (Gender Effects)

Males and females did not differ in narcissism propensity, self-disgust, and self-esteem (Table 3).

For gaze duration, the ANOVA revealed significant main effects for Condition (*F*(1,65) = 8.52, *p* = 0.005, *partη2* = 0.116) and Gender (*F*(1,65) = 13.49, *p* < 0.001, *partη2* = 0.172). The interaction Condition × Gender was not significant (*F*(1,65) = 0.15, *p* = 0.70, *partη2* = 0.002). The participants viewed their own face shorter than the unknown face (*d* = 0.372). On average, males (*M* = 81.33 s, *SD* = 4.39) looked longer at the faces (self and other) compared to females (*M* = 76.50 s, *SD* = 5.88, *t*(65) = 3.67, *p* < 0.001, *d* = 0.91).

For fixation count, the interaction Condition × Gender was significant (*F*(1, 65) = 5.50, *p* = 0.022, *partη2* = 0.078). However, the post-hoc comparisons were not significant (*p* > 0.060).

## 4. Discussion

This study investigated whether gaze parameters while viewing one’s face in the mirror are associated with the personality traits of self-esteem, self-disgust, and narcissism propensity. High self-esteem indicates that, on the whole, individuals are satisfied with themselves and are not overly critical [30,31]. In previous eye-tracking research, elevated self-esteem was associated with attentional biases towards self-faces [13]. Surprisingly, in the present investigation, higher self-esteem was associated with shorter—possibly less critical—viewing of the own face. It is possible that people with high self-esteem need less time to evaluate themselves (critically), while low self-esteem seems to be associated with a more thorough and more prolonged evaluation of one’s facial appearance.

Following this interpretation, the shorter viewing time of the other face associated with higher self-esteem would imply that individuals with high self-esteem are also less critical of others [32]. It is possible that in previous research [13] this viewing pattern could not be identified due to brief exposure times. The participants looked at the faces until they identified them. This took them less than one second on average. Additionally, in previous research, faces were not viewed freely, and task demands (face identification) may have overruled a critical and thorough evaluation of the faces [13]. Future research needs to investigate the effect of exposure time and viewing tasks on gaze behavior concerning self-face and other-face.

Self-esteem did neither predict the number of fixations on self-face nor other-face. Many short fixations (i.e., hyperscanning) are commonly conducted when affective stimuli with negative valence and high intensity are explored [22,33]. In the present study, dynamic faces with neutral expressions were viewed, which do not induce high arousal, at least in non-clinical samples [19,20,21]. While self-esteem predicted a more thorough evaluation of faces (longer viewing time), it was not associated with visual hyperscanning, which would have required elevated arousal. Taken together, the results suggest that low self-esteem is related to a thorough but calm evaluation of faces.

Earlier investigations indicated that the number of fixations on specific face areas differs between self-face and other-face viewing. Hoffmann et al. [8] observed that more fixations were conducted on the eyes when viewing unfamiliar faces than the own face. We refrained from using a similar analysis approach (an analysis of fixation counts for specific facial areas) because of insufficient spatial precision of the measuring procedure. There are slight interindividual differences in the position of facial features (e.g., location of eyes, mouth), which cannot be detected with sufficient spatial accuracy during mirror viewing. It would have been possible to control for the location of the eyes during mirror viewing by adjusting the chinrest for each participant until they see their eyes at a predefined mirror location. This procedure would, however, lead to self-face exposure before the eye-tracking experiment begins. Therefore, we focused on the gaze behavior for the face as a whole.

There was no relationship between NPI scores and gaze behavior. Narcissism propensity was neither associated with a more thorough (prolonged) viewing nor hyperscanning. These null-findings contrast our expectations and previous research, especially for self-face viewing [15]. In the present paradigm, participants viewed self-faces and other faces one after another and not simultaneously (e.g., as image pairs). It is possible that associations between narcissism propensity and viewing behavior did not show up because participants could not directly compare their faces with the faces of others. Furthermore, participants did not perform any task in which they could have compared their performance with the performance of others. Future studies should investigate whether narcissism propensity is associated with specific self-face viewing behaviors in competitive contexts (i.e., when comparing one’s own and others’ physical appearance).

The NPI assesses feelings of self-importance. However, narcissism has different facets. It has been suggested to distinguish between grandiose and vulnerable narcissism [34,35]. Vulnerable narcissistic individuals are anxious, defensive, and avoidant, while grandiose narcists are extraverted and self-satisfied [36]. Additionally, there are intrapersonal and interpersonal aspects of narcissism [37]. To maintain a grandiose self, people with a high narcissism propensity might, on the one hand, strive for feelings of uniqueness (intrapersonal) [38]. On the other hand, they can also devaluate others or strive for supremacy (interpersonal) [39]. Therefore, more differentiated NP measures should be used in the future, which can differentiate between these separable yet related expressions of narcissism that should prompt different styles of self-viewing [40].

Self-disgust also showed no association with viewing the own face. Self-disgust has been conceptualized as a dysfunctional personality trait [12,41] which typically has a very low level in mentally healthy individuals [41]. This was also the case in the present investigation. In contrast, Ypsilanti, Robson et al. [6] analyzed eye-tracking data from participants with mental health problems. This group with elevated self-disgust displayed avoidance of self-viewing. Therefore, the present null results do not contrast previous findings [5,6]. Future research should further investigate self-face viewing in conditions with elevated self-disgust, such as depression [2,42], disordered eating [43,44,45], and body dissatisfaction [45,46,47]. For example, body dysmorphic disorder (BDD) is associated with high self-disgust [47] but not with avoidance of self-viewing. Many patients with BDD even report spending a significant amount of time checking themselves in the mirror [45]. Mirror viewing paradigms can therefore contribute to a deeper understanding of self-viewing avoidance as well as excessive mirror viewing in eating disorders and BDD. For these disorders, mirror exposure is a commonly used therapy component [48].

We also investigated possible gender effects on face-viewing behavior. In general, males, as well as females, spent the majority of the 90 s-viewing time on the faces. Thus, there was no indication of face avoidance. Male participants were characterized by a longer total viewing time. They showed a longer gaze duration for both the own face and the other face. Previous studies have already demonstrated that face exploration dynamics (for other faces) differentiate men and women [49,50]. In an investigation by Coutrot et al. [49], the participants (203 males and 202 females) watched videos of actors. Male participants showed longer fixation durations for the faces than female participants. However, this difference was small. Overall, face viewing has many functions and serves multiple purposes (e.g., aiding speech perception [51], emotion recognition [52], person identification [53], or affective evaluation [15]). Moreover, context factors (e.g., cultural, social) have to be considered as well [54]. It needs further investigation whether differences in face-viewing intentions might have contributed to the observed gender difference in gaze duration.

It is plausible that several limitations might have influenced the results obtained. We studied a convenience sample, and the participants were healthy, predominantly young students. Therefore, the present results cannot be generalized to other samples. Furthermore, the study did not differentiate between different aspects of narcissism propensity.

## 5. Conclusions

This study is the first eye-tracking investigation on self-face viewing in a mirror. Gaze duration was associated with self-esteem. People with lower self-esteem viewed faces longer and possibly more critical. Furthermore, males looked longer at the faces than females. These insights contribute to a deeper understanding of mirror viewing behavior which is needed to improve and evaluate mirror exposure therapy in the future.

## Figures and Tables

**Table 1:** Pearson correlations between gaze duration and personality traits.

	Gaze Duration		Fixation Count	
	*R*	*p*	*r*	*p*
**Self-face**				
NPI	−0.080	0.517	0.150	0.222
QASD	0.114	0.356	0.042	0.734
RSES	−0.365 *	0.002	0.135	0.272
**Other-face**				
NPI	−0.171	0.166	−0.043	0.729
QASD	0.092	0.458	−0.094	0.449
RSES	−0.307 *	00.012	0.251 *	0.040

NPI: Narcissistic Personality Inventory; QASD: Questionnaire for the Assessment of Self-Disgust; RSES: Rosenberg Self-Esteem Scale; * *p* < 0.05.

**Table 2:** Regression analyses for gaze duration.

	*B* (CI)	*SE B*	β	*p*
**Self-face**				
NPI	−0.30 (−2.62, 2.02)	1.16	−0.033	0.796
QASD	−2.87 (−6.87, 1.14)	2.01	−0.254	0.157
RSES	−7.06 (−11.32, −2.80)	2.13	−0.545	0.002
**Other-face**				
NPI	−1.23 (−3.42, 0.96)	1.10	−0.148	0.266
QASD	−1.40 (−5.05, 2.25)	1.83	−0.141	0.445
RSES	−4.75 (−8.63, −0.87)	1.94	−0.416	0.017

NPI: Narcissistic Personality Inventory; QASD: Questionnaire for the Assessment of Self-Disgust; RSES: Rosenberg Self-Esteem Scale; B: unstandardized beta; CI: 95% confidence interval; SE: standard error; β: standardized beta.

**Table 3:** Descriptive statistics and gender comparisons.

	All M (SD)	Males M (SD)	Females M (SD)	T_66_ (p)	d
RSES	2.12 (0.51)	2.04 (0.54)	2.18 (0.48)	1.11 (0.812)	0.27
NPI	2.48 (0.72)	2.69 (0.73)	2.32 (0.69)	2.09 (0.204)	0.51
QASD	0.66 (0.58)	0.74 (0.63)	0.60 (0.54)	0.94 (0.699)	0.23
**Self-face**					
Gaze duration	77.75 (6.58)	80.70 (5.27)	75.56 (6.66)	3.43 (0.006) *	0.84
Fixation counts	127.49 (36.65)	134.45 (41.46)	122.31 (32.21)	1.36 (0.715)	0.33
**Other-face**					
Gaze duration	79.37 (5.83)	82.05 (4.77)	77.44 (5.80)	3.45 (0.007) *	0.86
Fixation counts	129.75 (44.36)	124.71 (44.87)	133.36 (44.21)	0.79 (0.436)	0.19

NPI: Narcissistic Personality Inventory; QASD: Questionnaire for the Assessment of Self-Disgust; RSES: Rosenberg Self-Esteem Scale; gaze duration in seconds; d: Cohen’s d; Asterisks: Bonferroni–Holm corrected *p* < 0.05.

## Data Availability

The data presented in this study are openly available in the Open Science Framework (OSF): https://osf.io/fmv5s/ (accessed on 10 August 2021).
